# Symptoms of Insomnia and Sleep Duration and Their Association with Incident Strokes: Findings from the Population-Based MONICA/KORA Augsburg Cohort Study

**DOI:** 10.1371/journal.pone.0134480

**Published:** 2015-07-31

**Authors:** A. Katharina Helbig, Doris Stöckl, Margit Heier, Karl-Heinz Ladwig, Christa Meisinger

**Affiliations:** 1 Institute of Epidemiology II, Helmholtz Zentrum München, German Research Center for Environmental Health (GmbH), Neuherberg, Germany; 2 Department for Psychosomatic Medicine and Psychotherapy, Klinikum Rechts der Isar, Technical University of Munich, Munich, Germany; Oasi Research Institute, ITALY

## Abstract

**Objective:**

To examine the relationship between symptoms of insomnia and sleep duration and incident total (non-fatal plus fatal) strokes, non-fatal strokes, and fatal strokes in a large cohort of men and women from the general population in Germany.

**Methods:**

In four population-based MONICA (monitoring trends and determinants in cardiovascular disease)/KORA (Cooperative Health Research in the Region of Augsburg) surveys conducted between 1984 and 2001, 17,604 men and women (aged 25 to 74 years) were asked about issues like sleep, health behavior, and medical history. In subsequent surveys and mortality follow-ups, incident stroke cases (cerebral hemorrhage, ischemic stroke, transient ischemic attack, unknown stroke type) were gathered prospectively until 2009. Sex-specific hazard ratios (HR) and their 95% confidence intervals (CI) were estimated using sequential Cox proportional hazards regression models.

**Results:**

During a mean follow-up of 14 years, 917 strokes (710 non-fatal strokes and 207 fatal strokes) were observed. Trouble falling asleep and difficulty staying asleep were not significantly related to any incident stroke outcome in either sex in the multivariable models. Among men, the HR for the association between short (≤5 hours) and long (≥10 hours) daily sleep duration and total strokes were 1.44 (95% CI: 1.01–2.06) and 1.63 (95% CI: 1.16–2.29), after adjustment for basic confounding variables. As for non-fatal strokes and fatal strokes, in the analyses adjusted for age, survey, education, physical activity, alcohol consumption, smoking habits, body mass index, hypertension, diabetes, and dyslipidemia, the increased risks persisted, albeit somewhat attenuated, but no longer remained significant. Among women, in the multivariable analyses the quantity of sleep was also not related to any stroke outcome.

**Conclusion:**

In the present study, symptoms of insomnia and exceptional sleep duration were not significantly predictive of incident total strokes, non-fatal strokes, and fatal strokes in either sex.

## Introduction

Strokes are one of the leading causes of severe disability [1], morbidity [2], and mortality [3] worldwide. According to estimates from the Global Burden of Disease Study, 16.9 million individuals suffered a first stroke in the year 2010. In the same year, 5.9 million stroke-related deaths occurred [4]. Besides the serious effects for the individuals concerned, their families, and their friends, strokes can cause considerable societal costs. For Germany, projections up to the year 2025 yielded expected direct costs of 108.6 billion EUR for first ischemic strokes alone [5].

The probability of suffering a stroke increases with age [6]. Due to the almost worldwide trend of aging populations [7], it is estimated that the number of individuals suffering from a stroke will continue to increase in the future. Various studies have demonstrated that primary prevention, for instance, living a healthy lifestyle, can reduce the risk of strokes [8–10]. Therefore, identifying modifiable risk factors is of particular interest to raise general awareness so that, ideally, preventive measures can be undertaken. Although there have been many advances in determining various stroke-related risk factors [8, 9], there are differing results regarding disturbed sleep [11, 12]. Moreover, whereas short and long sleep duration is well established as a risk factor for all-cause mortality [13], the association between exceptional sleep duration and stroke-related mortality is less clear [14].

Symptoms of insomnia, such as trouble falling asleep and difficulty staying asleep, affect approximately one-third of the German general population [15] and seem to be one of the most frequent health-related complaints in adults [16]. Sleep is an important factor for biological recovery functions [17]. Sleep deprivation has been shown to be associated with increased heart rate and decreased heart rate variability [18], raised inflammatory and proinflammatory markers [19], and the shortening of telomere length [20]. In addition, short sleep duration is related to unhealthy behavior, for example, the preference of energy-rich food and an increased number of meals [21], while on the other hand long sleep duration was found to be associated with poor physical and mental health [22]. All these factors might be linked to an increased risk of strokes. Thus, it could be hypothesized that symptoms of insomnia, as well as exceptional sleep duration, can be related to the incidence of strokes.

Therefore, the primary aim of this study was to investigate the influence of trouble falling asleep, difficulty staying asleep, and short and long sleep duration on the risk of developing strokes (non-fatal plus fatal) in men and women from the general population in Germany. As there is some evidence that the underlying mechanisms of developing a stroke, as opposed to dying from a stroke, might be different [23] and also for reasons of better comparability with previous research, a secondary goal was to examine whether the diverse sleep issues have different effects on suffering non-fatal strokes or fatal strokes, separately.

## Methods

### Study design and participants

The present post-hoc analyses were performed using data from the prospective, population-based cohort study, Monitoring Trends and Determinants in Cardiovascular Diseases (MONICA)/Cooperative Health Research in the Region of Augsburg (KORA) (details of the projects have been described previously [24–26]).

In brief, four independent cross-sectional surveys were conducted in 1984/85 (Survey (S) 1), 1989/90 (S2), 1994/95 (S3), and 1999/2001 (S4) among the general population of the region of Augsburg, Southern Germany. If an individual was chosen randomly for participation in more than one survey, only data from the respective baseline survey was considered for the analyses. Altogether, 17,604 individuals aged 25 to 74 years participated in at least one of the four surveys (response between 68% and 79%) [25–27]. Follow-up activities, including assessment of the vital status of the participants, causes of deaths, and incident diseases [26], were carried out until 2009.

Included in the analyses were data from participants who had not experienced a stroke prior to the baseline survey, with available information about the presence or absence of non-fatal strokes or fatal strokes during the follow-up period (n = 16,280). Data from 533 participants were excluded because of missing values in one or more of the variables required for the multivariable analyses (symptoms of insomnia: 25 missing values; other variables: 508 missing values). Thus, the final data set for analyses regarding symptoms of insomnia consisted of 7,911 men and 7,835 women aged 25 to 74 years. Owing to the fact that in the last survey (S4) information about sleep duration was not obtained, the basis for the analyses concerning the duration of average daily sleep was 6,157 men and 5,974 women in the same age range (sleep duration variable: 5 missing values).

The MONICA/KORA Augsburg Cohort Study was approved by the ethics committee of the Bavarian Medical Association (“Bayerische Landesärztekammer”) and the performances were realized in accordance with the Declaration of Helsinki. All individuals whose data were included in the analyses gave their written informed consent to taking part in the study and to having the information provided used for scientific purposes.

### Outcome variables

The study has the following three endpoints of interest: the respective first occurrences of 1) total (non-fatal plus fatal) strokes, 2) non-fatal strokes, and 3) fatal strokes from the time point of the baseline examination until the end of the follow-up on December 31, 2009.

Non-fatal strokes were assessed by asking “Have you ever had a stroke diagnosed by a physician?” in regular face-to-face interviews and postal surveys. Using data from participants’ hospital records and information gathered from their attending physicians, all potential stroke cases and the date of diagnosis were validated. During the validation process, non-fatal strokes were divided into embolic strokes, ischemic strokes, intracerebral hemorrhage, subarachnoid hemorrhage, transient ischemic attack/prolonged reversible ischemic neurological deficit, and unknown stroke type.

To ascertain fatal strokes, the survival status of all participants was regularly checked using information provided by population registries inside and outside the study area. Death certificates of deceased participants, preserved by local health authorities, were analyzed and the main causes of death extracted. As mentioned above, all information regarding fatal strokes was also checked by screening hospital records. Additionally, all hospital records were also screened to examine whether an individual who had died from a cause other than a stroke had suffered a stroke in the time between baseline/the last follow-up and the participant’s eventual death. The following three-digit International Classification of Diseases, Ninth Revision (ICD-9) codes were considered as strokes as cause of death: 430, 431, 433, 434, 436.

Regardless of the types of strokes, for the analyses all strokes were classified in total strokes, non-fatal strokes, and fatal strokes.

### Symptoms of insomnia and sleep duration

Symptoms of insomnia were assessed by self-report in a personal interview at baseline using two questions regarding trouble falling asleep (“Did you have trouble falling asleep?”) and difficulty staying asleep (“Did you have problems with sleeping through the night?”). Answer categories were on a Likert scale (often, sometimes, or almost never). To distinguish milder forms of disturbed sleep from more severe cases, both variables were binary coded, whereby participants were categorized as having a certain symptom of insomnia if they chose “often”and as having no difficulties if they chose “sometimes” or “almost never” as their response. Sleep duration was ascertained by using the question ‘‘How many hours a day do you usually sleep?”. The participants were encouraged to answer in whole numbers. As in former studies [28, 29], sleep duration was categorized in ≤5, 6, 7–8 (reference), 9, or ≥10 hours of daily sleep.

### Covariables

Baseline information about socio-demographic characteristics, lifestyle behavior, and health status of the study participants was obtained using self-administered questionnaires and face-to-face interviews by trained medical staff. The educational level of the participants was estimated by years of education, ≤11 years being classified as low educational level and >11 years as high educational level. Employment status was categorized as currently employed (full- or part-time) or unemployed. Physical activity during leisure time was assessed with a question concerning leisure-time sport activity in winter and in summer, respectively. Responses to these two questions were combined to illustrate the level of leisure-time physical activity (very active, moderately active, not very active, no sport [30]). For average daily alcohol consumption, sex-specific cut-off points were applied (0–39 and ≥40 grams (g) alcohol for men; 0–19 and ≥20 g alcohol for women) [31]. Participants were classified as current cigarette smoker (occasionally or regularly) or nonsmoker (individuals who had quit smoking previously or who had never smoked). History of physician-diagnosed diabetes and myocardial infarction were assessed by self-report. Angina pectoris was obtained using the categorization from Rose et al. [32]. Depressed mood was measured by means of the DEpression and EXhaustion scale (DEEX scale), a subscale of the von Zerssen checklist [33, 34]. Values ranged from 0 to 24, with higher values indicating a higher level of depressive symptomatology.

In addition to the extensive interviews, physical examinations were performed using standardized methods to gather information about baseline body mass index (BMI), dyslipidemia, and hypertension. To calculate the BMI of the participants, the measured weight in kilograms was divided by the square of height in meters. Dyslipidemia was defined as a ratio of total cholesterol divided by high-density lipoprotein (HDL) cholesterol ≥5 [25]. Methods used for measuring and analyzing total cholesterol and HDL cholesterol are expounded in detail in, e.g., [35] and [25]. Actual hypertension was defined as blood pressure ≥140/90 mmHg and/or the consumption of antihypertensive medication according to the Subcommittee of the World Health Organization and the International Society of Hypertension (WHO-ISH) [36], given that the participants were aware of being hypertensive. Blood pressure was measured with a random-zero sphygmomanometer in S1-S3 (Hawksley & Sons Ltd, Lancing, England) [37] and by use of an oscillometric digital blood pressure monitor (HEM-705CP, Omron Corporation, Tokyo, Japan) in S4 [38] in a sitting position. Antihypertensives that had been consumed during the past 7 days before study examination were recorded and classified by agents following the anatomic-therapeutic-chemical classification system. Antihypertensives were defined as any medication recommended by the German League for the Control of Hypertension (recently German Hypertension League) to lower the blood pressure (http://www.hochdruckliga.de; in the respective applicable version).

### Statistical analyses

The description of the baseline characteristics of the study participants was based on calculations of relative frequencies for categorical variables, means with corresponding standard deviations for normally distributed continuous variables, and medians with interquartile ranges for skewed data. Depending on the categorization of the variables and distribution of the data, differences were tested using chi-square test, Student’s t test, or Mann-Whitney U test stratified for men and women.

To predict the effect of the three sleep variables (trouble falling asleep, difficulty staying asleep, and sleep duration) on the risk of the first occurrence of the diverse stroke outcomes (total strokes, non-fatal strokes, and fatal strokes), four nested semi-parametric Cox proportional hazards regression models were estimated for each sleep variable [39].

Observation time was determined as starting at the time of baseline examination (S1-S4) and continued until date of stroke diagnosis, time of death (caused by a stroke or from a cause other than a stroke), time of last participation in one follow-up study, or the end of follow-up on December 31, 2009. For all participants for whom no follow-up was possible (e.g., prohibition of contact, unknown address), the values of the incident stroke variables were coded as missing. If the validation of potential stroke cases was not possible, participants were censored to an earlier time point. If this was not possible either, the values of the stroke variables were programmed as missing.

For analyses with non-fatal strokes as the outcome of interest, data from all participants with fatal strokes were excluded. The same procedure was carried out to examine fatal strokes as the dependent variable. Potential confounders were preselected based on the results of a prior literature review and bivariate associations with the stroke variables (p-value ≤0.2). All remaining covariables with significant associations were tested for interrelationships and for multicollinearity. More robust regression models were achieved by excluding the variables “employment status” and “heart symptoms/disease” owing to their lack of significance in the multivariable models in any stroke outcome. No violation of the proportional hazards assumption was assumed, because Schoenfeld residuals were independent of time and functions of time [40].

Four sequential models were estimated separately for each of the three sleep variables (trouble falling asleep, difficulty staying asleep, and sleep duration). Each of the first models (crude models) contained just one of the respective sleep variables. The second models (basic models) were, in addition to the models 1, adjusted for sex (men, women), age (continuous), and survey (1, 2, 3, 4). The third models (socio-demographic and lifestyle models) further included education (low, high), leisure-time physical activity level (very active, moderately active, not very active, no sport), alcohol consumption (men: 0–39 g/day, ≥40 g/day; women: 0–19 g/day, ≥20 g/day), and current smoking activity (yes, no). Finally, the full models (health status models) were additionally adjusted for BMI (continuous), hypertension (yes, no), diabetes (yes, no), and dyslipidemia (yes, no).

Estimates were presented as hazard ratios (HR) and their precision as 95% confidence intervals (CI). Interactions between each of the sleep variables and sex were tested; p-values ≤0.2 were considered to be statistically significant. Because there was evidence of an effect modification between sleep duration and sex (p-value = 0.0518), stratified analyses for men and women were performed.

As indicated in the literature, depressed mood seems to be associated with strokes and sleep disturbances [14, 41, 42]. Therefore, after the existing models 4 were augmented with the depressed mood variable, additional analyses were performed to examine its effect. Because the DEEX scale was not ascertained in survey S4, the analyses were realized with data from the first three surveys (S1-S3) (missing values in the depressed mood variable: n = 1,191).

Unless otherwise stated, p-values of ≤0.05 were deemed as statistically significant and all statistical tests were calculated two-tailed. The analyses were performed using the statistics program SAS (Statistical Analysis System, version 9.2, SAS Institute Inc., Cary, NC).

## Results

### Characteristics of study participants

During a mean follow-up time of 14 years, 710 non-fatal strokes (men: 455; women: 255) and 207 fatal strokes (men: 115; women: 92) were observed (crude incidence rate of total strokes: men: 5.3 per 1,000 person-years; women: 3.1 per 1,000 person-years).

Table 1 provides information about baseline characteristics of the study participants, stratified by the occurrence of an incident total stroke during the follow-up period for men and women separately.

**Table 1:** Prevalence (%) and mean (±standard deviation) of baseline population characteristics by sex and incident total strokes, men and women aged 25 to 74 years (N = 15,746).

	Men			Women		
	Incident total strokes			Incident total strokes		
	Yes	No	p-value	Yes	No	p-value
	(n = 570)	(n = 7,341)		(n = 347)	(n = 7,488)	
**Socio-demographic and lifestyle characteristics**						
Age (years)	58.6 (10.5)	47.7 (13.5)	< .0001	60.1 (10.1)	47.4 (13.2)	< .0001
Low educational level (%)	72.5	61.9	< .0001	88.2	77.3	< .0001
Employed^1^ (%)	48.3	73.4	< .0001	21.9	48.3	< .0001
Physically active >2 hours/week (%)	17.4	22.9	< .0001	11.0	13.8	< .0001
High consumption of alcohol (%)	33.9	30.4	0.0848	14.7	19.0	0.0442
Currently smoking (%)	29.3	32.2	0.1503	17.3	21.6	0.0582
**Health status**						
Body mass index (kg/m^2^)	28.5 (3.6)	27.1 (3.7)	< .0001	28.2 (4.9)	26.3 (4.9)	< .0001
Hypertension (%)	67.5	42.3	< .0001	61.1	30.1	< .0001
History of heart symptoms/disease^2^ (%)	9.1	5.6	0.0005	8.1	4.3	0.0009
Diabetes (%)	9.8	3.7	< .0001	13.0	2.9	< .0001
Dyslipidemia (%)	52.3	42.4	< .0001	34.6	16.2	< .0001
Depressed mood^3^ (score 0–24)	9.0 (5.0–12.0)	8.0 (5.0–11.0)	0.0677	9.0 (5.0–13.0)	10.0 (6.0–13.0)	0.1698
**Sleep disturbances and sleep duration**						
Trouble falling asleep (%)	11.9	8.3	0.0032	22.8	16.0	0.0008
Difficulty staying asleep (%)	21.8	15.2	< .0001	27.4	21.4	0.0083
Sleep duration^4^ (hours per 24 hours)			< .0001			0.0155
≤5	6.7	4.5		4.4	4.2	
6	11.6	15.2		16.0	11.9	
7–8	64.6	70.8		62.9	71.6	
9	9.7	6.1		11.3	8.1	
≥10	7.5	3.5		5.4	4.2	

^1^ Variable was available for 15,742 individuals.

^2^ Variable was available for 15,732 individuals.

^3^ Variable was available for 10,945 individuals. Median and interquartile range.

^4^ Variable was available for 12,131 individuals.

When compared with participants who did not suffer a first total stroke since the beginning of the study, individuals of both sexes who experienced a subsequent stroke reported more often trouble falling asleep and difficulty staying asleep at baseline examination. Men and women with a short (≤5 hours) or long (≥10 hours) usual daily sleep duration also developed more frequently a first total stroke during the study time than participants who slept 7–8 hours daily.

Furthermore, among those who suffered a stroke during the study time, a higher proportion of the participants were male, and they were on average significantly older than those who did not. They were more likely to have a lower educational level and were less physically active in their leisure time. Moreover, they often had a higher BMI and an impaired health status, with, for example, a higher proportion of hypertension, diabetes, or dyslipidemia.

### Symptoms of insomnia and incident strokes

The unadjusted and multivariable adjusted HR for the first occurrence of total strokes, non-fatal strokes, and fatal strokes since baseline examination by symptoms of insomnia are presented in Table 2 for men and in Table 3 for women.

**Table 2:** Hazard ratios and 95% confidence intervals for the incidence of total strokes (N = 7,911), non-fatal strokes (N = 7,796), and fatal strokes (N = 7,456) according to self-reported symptoms of insomnia, men aged 25 to 74 years.

Men	Model 1	Model 2	Model 3	Model 4
	HR (95% CI)	HR (95% CI)	HR (95% CI)	HR (95% CI)
**Trouble falling asleep**				
Total strokes (n = 68^1^)	**1.59 (1.24–2.05)**	1.25 (0.97–1.62)	1.21 (0.93–1.56)	1.17 (0.91–1.52)
Non-fatal strokes (n = 52^1^)	**1.53 (1.14–2.04)**	1.20 (0.90–1.60)	1.16 (0.86–1.54)	1.12 (0.84–1.51)
Fatal strokes (n = 16^1^)	**1.95 (1.15–3.31)**	1.53 (0.90–2.59)	1.50 (0.88–2.56)	1.44 (0.84–2.48)
**Difficulty staying asleep**				
Total strokes (n = 124^1^)	**1.78 (1.46–2.17)**	1.09 (0.89–1.33)	1.09 (0.89–1.33)	1.06 (0.87–1.30)
Non-fatal strokes (n = 99^1^)	**1.79 (1.43–2.23)**	1.11 (0.88–1.39)	1.11 (0.88–1.39)	1.08 (0.86–1.35)
Fatal strokes (n = 25^1^)	**1.84 (1.18–2.86)**	1.02 (0.65–1.59)	1.02 (0.66–1.60)	1.00 (0.64–1.56)

Abbreviation: HR: hazard ratio, CI: confidence interval, N: Size of the underlying study population with regard to the particular stroke outcome.

Model 1: crude model (unadjusted).

Model 2: adjusted for age (continuous) and survey (1, 2, 3, 4).

Model 3: “Socio-demographic and lifestyle model” was, in addition to model 2, further adjusted for education (low, high), physical activity (>2 h/week, 1 h/week (regular), 1 h/week (irregular), no sport), alcohol consumption (men: 0–39 g/day, ≥40 g/day; women: 0–19 g/day, ≥20 g/day), current smoking activity (yes, no).

Model 4: The full model (“health status model”) was, in addition to model 3, adjusted for BMI (continuous), hypertension (yes, no), diabetes (yes, no), dyslipidemia (yes, no).

^1^ Number of incident stroke cases.

**Table 3:** Hazard ratios and 95% confidence intervals for the incidence of total strokes (N = 7,835), non-fatal strokes (N = 7,743), and fatal strokes (N = 7,580) according to self-reported symptoms of insomnia, women aged 25 to 74 years.

Women	Model 1	Model 2	Model 3	Model 4
	HR (95% CI)	HR (95% CI)	HR (95% CI)	HR (95% CI)
**Trouble falling asleep**				
Total strokes (n = 79^1^)	**1.75 (1.36–2.25)**	0.91 (0.70–1.17)	0.90 (0.70–1.16)	0.92 (0.71–1.18)
Non-fatal strokes (n = 57^1^)	**1.72 (1.28–2.31)**	0.93 (0.69–1.25)	0.91 (0.68–1.23)	0.92 (0.68–1.24)
Fatal strokes (n = 22^1^)	**1.90 (1.18–3.07)**	0.84 (0.52–1.36)	0.85 (0.52–1.38)	0.86 (0.53–1.39)
**Difficulty staying asleep**				
Total strokes (n = 95^1^)	**1.63 (1.29–2.07)**	0.85 (0.67–1.09)	0.86 (0.68–1.09)	0.87 (0.68–1.10)
Non-fatal strokes (n = 71^1^)	**1.67 (1.27–2.19)**	0.89 (0.67–1.17)	0.89 (0.68–1.18)	0.89 (0.68–1.18)
Fatal strokes (n = 24^1^)	1.56 (0.98–2.49)	0.75 (0.47–1.19)	0.75 (0.47–1.19)	0.75 (0.47–1.20)

Abbreviation: HR: hazard ratio, CI: confidence interval, N: Size of the underlying study population with regard to the particular stroke outcome.

Model 1: crude model (unadjusted).

Model 2: adjusted for age (continuous) and survey (1, 2, 3, 4).

Model 3: “Socio-demographic and lifestyle model” was, in addition to model 2, further adjusted for education (low, high), physical activity (>2 h/week, 1 h/week (regular), 1 h/week (irregular), no sport), alcohol consumption (men: 0–39 g/day, ≥40 g/day; women: 0–19 g/day, ≥20 g/day), current smoking activity (yes, no).

Model 4: The full model (“health status model”) was, in addition to model 3, adjusted for BMI (continuous), hypertension (yes, no), diabetes (yes, no), dyslipidemia (yes, no).

^1^ Number of incident stroke cases.

Regarding total strokes, significant associations between trouble falling asleep and difficulty staying asleep occurred in the crude models (models 1), but attenuated to insignificance in models 2–4 in both sexes. The crude HR for fatal strokes in men who reported trouble falling asleep and difficulty staying asleep were 1.95 (95% CI: 1.15–3.31) and 1.84 (95% CI: 1.18–2.86); for women they were 1.90 (95% CI: 1.18–3.07) and 1.56 (95% CI: 0.98–2.49) (models 1). In the models adjusted for age and survey (models 2), significant associations were no longer observed.

### Sleep duration and incident strokes

Table 4 shows the associations between self-reported sleep duration and total strokes, non-fatal strokes, and fatal strokes for men; Table 5 presents the corresponding results for women.

**Table 4:** Hazard ratios and 95% confidence intervals for the incidence of total strokes (N = 6,157), non-fatal strokes (N = 6,048), and fatal strokes (N = 5,758) according to self-reported sleep duration (hours per 24 hours), men aged 25 to 74 years.

Men	Model 1	Model 2	Model 3	Model 4
	HR (95% CI)	HR (95% CI)	HR (95% CI)	HR (95% CI)
**Total strokes**				
**Sleep duration**				
≤5 (n = 34^1^)	**1.84 (1.29–2.62)**	**1.44 (1.01–2.06)**	1.41 (0.99–2.01)	1.36 (0.95–1.94)
6 (n = 59^1^)	0.87 (0.66–1.15)	0.93 (0.70–1.23)	0.94 (0.71–1.24)	0.92 (0.70–1.22)
7–8 (n = 328^1^)	1.00	1.00	1.00	1.00
9 (n = 49^1^)	**2.16 (1.60–2.92)**	1.14 (0.84–1.54)	1.08 (0.80–1.47)	1.05 (0.78–1.43)
≥10 (n = 38^1^)	**3.25 (2.32–4.55)**	**1.63 (1.16–2.29)**	**1.50 (1.06–2.11)**	1.38 (0.98–1.94)
**Non-fatal strokes**				
**Sleep duration**				
≤5 (n = 24^1^)	**1.65 (1.09–2.51)**	1.35 (0.89–2.05)	1.32 (0.87–2.00)	1.29 (0.85–1.96)
6 (n = 49^1^)	0.90 (0.67–1.22)	0.95 (0.70–1.29)	0.96 (0.71–1.30)	0.95 (0.70–1.29)
7–8 (n = 262^1^)	1.00	1.00	1.00	1.00
9 (n = 37^1^)	**2.07 (1.47–2.92)**	1.13 (0.79–1.60)	1.07 (0.76–1.52)	1.05 (0.74–1.49)
≥10 (n = 27^1^)	**2.92 (1.96–4.34)**	**1.52 (1.02–2.27)**	1.38 (0.92–2.06)	1.27 (0.85–1.91)
**Fatal strokes**				
**Sleep duration**				
≤5 (n = 10^1^)	**2.75 (1.41–5.35)**	**2.04 (1.05–3.98)**	**2.06 (1.05–4.02)**	1.89 (0.96–3.73)
6 (n = 10^1^)	0.73 (0.37–1.42)	0.77 (0.40–1.51)	0.78 (0.40–1.51)	0.76 (0.39–1.48)
7–8 (n = 66^1^)	1.00	1.00	1.00	1.00
9 (n = 12^1^)	**2.69 (1.45–4.98)**	1.13 (0.60–2.10)	1.09 (0.58–2.03)	1.02 (0.54–1.91)
≥10 (n = 11^1^)	**4.77 (2.52–9.06)**	**1.97 (1.03–3.77)**	**1.97 (1.03–3.77)**	1.72 (0.89–3.33)

Abbreviation: HR: hazard ratio, CI: confidence interval, N: Size of the underlying study population with regard to the particular stroke outcome, h: hour.

Model 1: crude model (unadjusted).

Model 2: adjusted for age (continuous) and survey (1, 2, 3, 4).

Model 3: “Socio-demographic and lifestyle model” was, in addition to model 2, further adjusted for education (low, high), physical activity (>2 h/week, 1 h/week (regular), 1 h/week (irregular), no sport), alcohol consumption (men: 0–39 g/day, ≥40 g/day; women: 0–19 g/day, ≥20 g/day), current smoking activity (yes, no).

Model 4: The full model (“health status model”) was, in addition to model 3, adjusted for BMI (continuous), hypertension (yes, no), diabetes (yes, no), dyslipidemia (yes, no).

^1^ Number of incident stroke cases.

**Table 5:** Hazard ratios and 95% confidence intervals for the incidence of total strokes (N = 5,974), non-fatal strokes (N = 5,885), and fatal strokes (N = 5,745) according to self-reported sleep duration (hours per 24 hours), women aged 25 to 74 years.

Women	Model 1	Model 2	Model 3	Model 4
	HR (95% CI)	HR (95% CI)	HR (95% CI)	HR (95% CI)
**Total strokes**				
**Sleep duration**				
≤5 (n = 14^1^)	1.40 (0.81–2.40)	0.68 (0.40–1.18)	0.67 (0.39–1.16)	0.68 (0.40–1.18)
6 (n = 51^1^)	**1.66 (1.22–2.25)**	1.21 (0.89–1.65)	1.19 (0.87–1.62)	1.25 (0.91–1.70)
7–8 (n = 200^1^)	1.00	1.00	1.00	1.00
9 (n = 36^1^)	**1.64 (1.15–2.34)**	1.17 (0.82–1.68)	1.17 (0.82–1.67)	1.09 (0.76–1.57)
≥10 (n = 17^1^)	**1.72 (1.05–2.82)**	1.09 (0.66–1.79)	1.08 (0.66–1.79)	0.91 (0.55–1.51)
**Non-fatal strokes**				
**Sleep duration**				
≤5 (n = 10^1^)	1.36 (0.72–2.59)	0.68 (0.36–1.30)	0.67 (0.35–1.28)	0.69 (0.36–1.31)
6 (n = 34^1^)	**1.52 (1.05–2.21)**	1.13 (0.78–1.65)	1.11 (0.76–1.61)	1.15 (0.79–1.68)
7–8 (n = 146^1^)	1.00	1.00	1.00	1.00
9 (n = 26^1^)	**1.63 (1.08–2.48)**	1.20 (0.79–1.83)	1.20 (0.79–1.82)	1.12 (0.74–1.71)
≥10 (n = 13^1^)	**1.81 (1.03–3.20)**	1.17 (0.66–2.08)	1.17 (0.66–2.07)	0.98 (0.55–1.74)
**Fatal strokes**				
**Sleep duration**				
≤5 (n = 4^1^)	1.48 (0.53–4.07)	0.61 (0.22–1.69)	0.60 (0.22–1.67)	0.61 (0.22–1.69)
6 (n = 17^1^)	**2.06 (1.19–3.55)**	1.43 (0.82–2.47)	1.42 (0.82–2.46)	1.55 (0.89–2.70)
7–8 (n = 54^1^)	1.00	1.00	1.00	1.00
9 (n = 10^1^)	1.70 (0.87–3.35)	1.05 (0.53–2.06)	1.04 (0.52–2.05)	1.00 (0.50–1.98)
≥10 (n = 4^1^)	1.51 (0.55–4.18)	0.87 (0.31–2.43)	0.88 (0.31–2.45)	0.72 (0.26–2.06)

Abbreviation: HR: hazard ratio, CI: confidence interval, N: Size of the underlying study population with regard to the particular stroke outcome, h: hour.

Model 1: crude model (unadjusted).

Model 2: adjusted for age (continuous) and survey (1, 2, 3, 4).

Model 3: “Socio-demographic and lifestyle model” was, in addition to model 2, further adjusted for education (low, high), physical activity (>2 h/week, 1 h/week (regular), 1 h/week (irregular), no sport), alcohol consumption (men: 0–39 g/day, ≥40 g/day; women: 0–19 g/day, ≥20 g/day), current smoking activity (yes, no).

Model 4: The full model (“health status model”) was, in addition to model 3, adjusted for BMI (continuous), hypertension (yes, no), diabetes (yes, no), dyslipidemia (yes, no).

^1^ Number of incident stroke cases.

Total strokes are associated with long daily sleep duration only until model 3 in men. Among women, in the multivariable analyses (models 2–4), the amount of sleep was not related to any stroke outcome. Concerning non-fatal strokes, the relationship with long daily sleep duration among men remained significant in the basic model (model 2), but not after a further adjustment for education, physical activity, alcohol consumption, and smoking habits (model 3). Among men, in the socio-demographic and lifestyle model (model 3), short and long daily sleep duration was significantly associated with the development of fatal strokes (HR 2.06, 95% CI: 1.05–4.02 and HR 1.97, 95% CI: 1.03–3.77). After an additional adjustment for BMI, hypertension, diabetes, and dyslipidemia (model 4), this relationship was no longer significant (HR 1.89, 95% CI: 0.96–3.73 and HR 1.72, 95% CI: 0.89–3.33).

### Additional analyses

In the full models (models 4) additionally adjusted for depressed mood, a significant association was found between short sleep duration and fatal strokes (HR 2.29, 95% CI: 1.13–4.65) in men only (see S1 Table).

## Discussion

In the presented analyses, no associations were observed between trouble falling asleep or difficulty staying asleep and the risk of developing any stroke outcome in either sex. Among women, in the multivariable analyses the quantity of sleep was also not related to any stroke outcome. However, among men, sleeping ≤5 hours or ≥10 hours daily significantly increased the risk of developing total strokes, non-fatal strokes, and fatal strokes compared to those sleeping 7–8 hours in the unadjusted analyses. These increased risks persisted, albeit somewhat attenuated, but no longer remained significant after adjustment for a wide range of covariates, including socio-demographic aspects, health status, several diseases, and medical history.

The relationship between symptoms of insomnia and sleep duration and the development of strokes has been investigated before [43, 44]. However, many studies had cross-sectional study designs [45–51], and some others regarded specific populations, for instance, middle-aged male employees at a Japanese factory [52]. Prospective population-based studies revealed varying results.

Two studies using Taiwan’s National Health Insurance Research Database found a higher risk of developing ischemic strokes [53] and total strokes [11] in individuals with insomnia, compared to controls without insomnia. In accordance with our results, Westerlund et al. [12] observed no association between insomnia symptoms and incident total strokes in their prospective Swedish National March Cohort Study comprising 41,192 adults. These results were confirmed by a Norwegian study in which insomnia was related to incident non-fatal strokes in the unadjusted analyses, but not after taking into account all covariates [54]. Besides other effects resulting from the different geographic, social, and cultural backgrounds of the Asian and European studies, a further possible reason for these disparate results might be the varying definitions of insomnia. In the two Asian studies, insomnia was defined as a medical diagnosis of insomnia according to the clinical modification of the ICD-9. In the present study and in the two other European studies, however, information about perceived symptoms of insomnia was obtained by self-report using standardized questionnaires. Further, it may be that only insomnia severe enough to attract attention in an inpatient or outpatient setting led to finding an association with the development of strokes, as in the two Asian studies based on inpatient and outpatient claims data. Moreover, the database of these studies did not encompass important possible confounders, including BMI, physical activity, smoking habits, and alcohol consumption [11, 53]. Therefore, it is also conceivable that the restrictions in the confounder adjustment are responsible for the significant associations between insomnia and strokes in the Asian studies. This presumption is further supported by a study from Elwood et al. [55] consisting of 1,986 men aged 55 to 69 years, in which frequent insomnia was significantly associated with incident ischemic strokes. Even in this study, an adjustment for variables like physical activity, chronic diseases, or medication intake was not performed.

In the Whitehall II Cohort Study, which included 9,098 participants, having unusually restless, disturbed nights was associated with cardiovascular disease mortality only in women [56]. Another study, using data from a French cohort study with 16,989 participants, showed neither trouble falling asleep nor difficulty staying asleep as significantly related to cardiovascular disease mortality among men. Because of small numbers of cases in each category for cause-specific mortality among women in this study, these analyses were realized only for men [57]. Stone and colleagues [58] reported in their Study of Osteoporotic Fractures comprising 8,101 women aged 69 years and over a significantly positive association between self-reported daily napping and cardiovascular disease mortality. Yet none of these studies performed separate analyses for stroke-specific mortality.

Regarding sleep duration, von Ruesten et al. [59] observed a relationship between both short and long daily sleep duration and incident strokes in their European Prospective Investigation into Cancer and Nutrition (EPIC)-Potsdam Study. However, Westerlund et al. [12] and Amagai et al. [60] found no harmful effects of the amount of sleep on incident strokes; other studies observed only one exceptional sleep duration category to be predictive of them [29, 61–63].

Studies with regard to fatal strokes as the outcome of interest are scarce and widely lacking for the European region [14, 63–67]. In the present study, a U-shaped relationship between sleep duration and fatal strokes emerged in the models adjusted for age, survey, socio-demographic, and lifestyle variables among men (models 1–3). However, after additional adjustment for BMI, hypertension, diabetes, and dyslipidemia (model 4), the association no longer remained significant. Unfortunately, a responsible interpretation of the data for women was not possible due to the small number of fatal stroke cases among them. In the literature, the authors of a recently published study observed a U-shaped relationship between sleep duration and fatal strokes in their population-based cohort of 63,257 Chinese adults aged 45 to 74 years, even after adjustment for all covariables [14]. In three other studies, conducted in Japan, Los Angeles/Hawaii, and China, each comprising more than 95,000 participants, only long sleep duration was significantly associated with a higher risk of stroke mortality in both sexes [65–67]. Therefore, it seems that in the present study, maybe the power to detect differences in the risk of fatal strokes between men with short or long sleep duration compared to those sleeping 7–8 hours daily was merely too low. In a smaller Japanese study from Amagai et al. [64] with about 11,000 individuals, a sleep duration of 6 hours was related to fatal strokes only in women. However, in this study, an adjustment for confounders like physical activity or diabetes was lacking. In another recently published study from Leng et al. [63] consisting of 9,692 men and women aged 42 to 81 years from the British population, long sleep duration was associated with fatal strokes among women only. A possible explanation for the differing results regarding the association between long sleep duration and risk of strokes may be the predominance of older participants in this and the other abovementioned studies. With advancing age, the probability of experiencing health impairments increases generally [68]. In a study from Kakizaki et al. [69], the authors observed that the relationship between long sleep duration and fatal strokes was strongest in participants with limited physical function and poorer self-rated general health status. Other possible explanatory approaches could involve, for example, different assessments and categorizations of sleep duration variables and covariates, and varying follow-up lengths.

Short sleep duration and early-morning awakening were commonly associated with mood disorders, painful conditions, and other medical disorders [70–72]. However, insomnia with objectively measured short sleep duration is suspected to be the most biologically severe phenotype of insomnia disorder [73]. In the literature, there is also evidence of a combined effect of symptoms of insomnia and short sleep duration on the risk of cardiovascular diseases [12, 74]. In the study of Westerlund et al. [12], individuals with short sleep who reported frequent symptoms of insomnia had the highest risk of overall cardiovascular events, while no risk increases were found due to either exposure alone (albeit not significant in the full models). Also in a Dutch population-based cohort study, the authors observed that individuals with short sleep duration and poor sleep quality had a 63% higher risk of cardiovascular diseases compared to individuals with 7 hours of daily sleep and good sleep quality, even after adjustments for all covariates [74]. Unfortunately, stroke-specific analyses were not carried out. Therefore, maybe the simultaneous presence of symptoms of insomnia and short sleep duration leads to a relationship with a higher propensity for strokes.

Interestingly, in the present study in the analyses additionally adjusted for depressed mood, in men the positive association between short sleep duration and fatal strokes became significant in the fully adjusted models (HR 2.29, 95% CI: 1.13–4.65 versus HR 1.89, 95% CI: 0.96–3.73) (see S1 Table). These results would suggest that the influence of sleep duration on the risk of the development of strokes is increased by the additional adjustment for depression. Yet the depressed mood variable shows a high number of missing values (n = 1,191), which could be due to the notable sensitivity of questions regarding the psychopathological state of an individual [75, 76]. Thus, it would be preferable if future research could help to clarify the role of mental health in the association between sleep and risk of strokes.

Furthermore, the association between sleep and strokes seems to be different between men and women. In the present study and in the literature (e.g., [77]), the proportion of men who suffered from strokes is higher in comparison to women, and it is known that sex and gender differences are responsible for different sleep behavior in both sexes [78]. Thus, it would be desirable that further studies examine the influence of sex- and gender-related aspects on the relationship between sleep and strokes.

The present study has several limitations and strengths that should be mentioned.

Firstly, the follow-up was not complete for all participants of the respective baseline surveys (S1-S4), which might have induced a selection bias [79].

Secondly, because the screening question concerning strokes was answered by self-report of the participants, it is conceivable that not all actual stroke cases were detected. Because some strokes are only moderately pronounced and there is a decline of stroke symptoms in some cases (transient ischemic attacks) [80], it may be possible that the number of stroke cases was underestimated in the present study. However, in our study, during a mean follow-up time of 14 years, 917 first-ever strokes were observed, and the crude incidence rate was roughly comparable with the incidence rates in similar studies [6, 81]. Even so, although the study population was large, the number of stroke cases divided by sleep duration categories was small, especially in the nonreference sleep duration categories, and resulted therefore in wide confidence intervals.

Thirdly, symptoms of insomnia were commonly assessed using established screening tools like the Insomnia Severity Index [82–84] or the Pittsburgh Sleep Quality Index [85, 86]. In the present study, symptoms of insomnia were obtained by means of two standardized questions concerning trouble falling asleep and difficulty staying asleep. These two questions were implemented in the initial World Health Organization’s MONICA Augsburg Study in the years 1984/85 [24, 25] and retained until 2001 for reasons of better comparability of the data. The above mentioned screening tools had not been published at the time of the first MONICA Augsburg Study. Nevertheless, the frequency of insomnia symptoms in the present study and in the literature is quite similar [15]. A further limitation of this study is the lack of information about the respective causes of insomnia symptoms, whether the symptoms were acute or chronic, and about possible changes in insomnia symptoms and sleep duration during the study period, because in the present study the current sleep-related issues were assessed only in the respective baseline surveys. Although some prospective studies indicate that symptoms of insomnia often tend to be persistent and become chronic [87, 88], a large variability can also be recorded [89, 90]. Therefore, it is of particular interest that future studies perform repeated assessments of sleep issues in short intervals during the follow-up period to take into consideration the natural course of insomnia and sleep duration over time [89]. According to the literature [91], self-reported sleep duration is moderately correlated with sleep duration measured by an actigraph. Yet the authors found that subjective reports of sleep duration were biased due to systematic overreporting by the participants. Additionally, we asked the participants only to report the frequency of insomnia symptoms. The literature reports a considerable interindividual variability with regard to the susceptibility of individuals to the effects of sleep disturbances [18]. Therefore, the individual impact of symptoms of insomnia or exceptional sleep duration could also be connected to the incidence of strokes.

Finally, we have included a wide range of covariables in the analyses, such as socio-demographic aspects, health status, several diseases, and medical history. Regrettably, in our study there is no information available on whether the participants suffer from sleep apnea. Sleep apnea occurs predominantly in men [92] and is commonly associated with cardiovascular conditions, like hypertension [93]. One assumption is that patients suffering from sleep apnea compensate for the disruption of sleep accompanying this syndrome by spending a longer period of time in bed or by long sleep duration [22, 94]. Therefore, it may be possible that the incidence of strokes is associated with aspects related to sleep apnea, and not with the tendency of long sleep duration by itself [22]. Further research should explore these issues.

The most important strengths of the present study are the prospective study design with high overall responses and a long follow-up length. Further positive considerations are the large population-representative database including the availability of information about several sleep aspects, stroke outcomes, and potential covariables, which enabled statistical analyses with complex multivariable methods. A further advantage is the physician-validated diagnosis of strokes. High-quality standards and controls [25, 26] also strengthened the results of the MONICA/KORA Augsburg Cohort Study.

## Conclusion

In conclusion, the results suggest that trouble falling asleep and difficulty staying asleep are not associated with incident total strokes, non-fatal strokes, or fatal strokes in either sex. There is an increased risk especially for fatal strokes in men with short and long sleep duration, albeit insignificant. Further research should explore the role of a combined effect of symptoms of insomnia and short sleep duration in association with incident strokes taking into account the sex- and gender-specific particularities regarding this issue.

## Supporting Information

S1 Table Hazard ratios and 95% confidence intervals of models 4 and models 4 plus depression for the incidence of total strokes, non-fatal strokes, and fatal strokes according to self-reported sleep disturbances, men and women aged 25 to 74 years. (PDF)
