# Supplementary material for: Symptoms of Insomnia and Sleep Duration and Their Association with Incident Strokes: Findings from the Population-Based MONICA/KORA Augsburg Cohort Study
Source: PLoS One. 2015 Jul 31;10(7):e0134480. doi: 10.1371/journal.pone.0134480 (PMC4521822; doi:10.1371/journal.pone.0134480)
Supplement: S1 Table — (PDF) [file pone.0134480.s001.pdf]

S 1 Table: Hazard ratios and 95% confidence intervals of models 4 and models 4 plus depression for the incidence of total strokes, non-fatal strokes, and fatal strokes according to self-reported sleep disturbances, men and women aged 25 to 74 years

	**Men**		**Women**	
	**Model 4**	**Model 4 + Depression**	**Model 4**	**Model 4 + Depression**
	HR (95% CI)	HR (95% CI)	HR (95% CI)	HR (95% CI)
**Trouble falling asleep**				
Total strokes	1.17 (0.91-1.52)	1.17 (0.86-1.58)	0.92 (0.71-1.18)	0.87 (0.63-1.21)
Non-fatal strokes	1.12 (0.84-1.51)	1.08 (0.76-1.53)	0.92 (0.68-1.24)	0.95 (0.65-1.39)
Fatal strokes	1.44 (0.84-2.48)	1.62 (0.88-3.00)	0.86 (0.53-1.39)	0.66 (0.34-1.26)
**Difficulty staying asleep**				
Total strokes	1.06 (0.87-1.30)	0.99 (0.78-1.26)	0.87 (0.68-1.10)	0.96 (0.71-1.30)
Non-fatal strokes	1.08 (0.86-1.35)	0.95 (0.72-1.26)	0.89 (0.68-1.18)	1.06 (0.75-1.51)
Fatal strokes	1.00 (0.64-1.56)	1.12 (0.68-1.84)	0.75 (0.47-1.20)	0.71 (0.39-1.29)
**Sleep duration**				
**≤5 hours**				
Total strokes	1.36 (0.95-1.94)	1.43 (0.98-2.10)	0.68 (0.40-1.18)	0.66 (0.33-1.30)
Non-fatal strokes	1.29 (0.85-1.96)	1.35 (0.86-2.12)	0.69 (0.36-1.31)	0.72 (0.33-1.55)
Fatal strokes	1.89 (0.96-3.73)	**2.29 (1.13-4.65)**	0.61 (0.22-1.69)	0.47 (0.11-1.99)
**6 hours**				
Total strokes	0.92 (0.70-1.22)	0.97 (0.72-1.29)	1.25 (0.91-1.70)	1.32 (0.92-1.88)
Non-fatal strokes	0.95 (0.70-1.29)	0.97 (0.70-1.34)	1.15 (0.79-1.68)	1.28 (0.83-1.95)
Fatal strokes	0.76 (0.39-1.48)	0.88 (0.45-1.72)	1.55 (0.89-2.70)	1.49 (0.78-2.85)
**7-8 hours**				
Total strokes	1.00	1.00	1.00	1.00
Non-fatal strokes	1.00	1.00	1.00	1.00
Fatal strokes	1.00	1.00	1.00	1.00
**9 hours**				
Total strokes	1.05 (0.78-1.43)	1.08 (0.77-1.51)	1.09 (0.76-1.57)	1.11 (0.74-1.67)
Non-fatal strokes	1.05 (0.74-1.49)	1.12 (0.77-1.64)	1.12 (0.74-1.71)	1.27 (0.80-2.00)
Fatal strokes	1.02 (0.54-1.91)	0.90 (0.44-1.86)	1.00 (0.50-1.98)	0.77 (0.32-1.85)
**≥10 hours**				
Total strokes	1.38 (0.98-1.94)	1.37 (0.94-2.01)	0.91 (0.55-1.51)	0.86 (0.47-1.56)
Non-fatal strokes	1.27 (0.85-1.91)	1.33 (0.86-2.07)	0.98 (0.55-1.74)	0.90 (0.45-1.79)
Fatal strokes	1.72 (0.89-3.33)	1.52 (0.71-3.24)	0.72 (0.26-2.06)	0.77 (0.23-2.53)

Abbreviation: HR: hazard ratio, CI: confidence interval.

Model 4: adjusted for age (continuous), survey (1, 2, 3, 4), education (low, high), physical activity (>2 h/week, 1 h/week (regular), 1 h/week (irregular), no sport), alcohol consumption (men: 0-39 g/day, ≥40 g/day; women: 0-19 g/day, ≥20 g/day), current smoking activity (yes, no), BMI (continuous), hypertension (yes, no), diabetes (yes, no), dyslipidemia (yes, no).

Model 4 + Depression: this model was, in addition to model 4, further adjusted for depression.
